# Masculinizing Effects of Chrysin-Loaded Poloxamer Micelles on Siamese Fighting Fish

**DOI:** 10.3390/vetsci8120305

**Published:** 2021-12-02

**Authors:** Nattakanwadee Khumpirapang, Tanongsak Sassa-deepaeng, Krit Suknuntha, Songyot Anuchapreeda, Siriporn Okonogi

**Affiliations:** 1 Department of Pharmaceutical Chemistry and Pharmacognosy, Faculty of Pharmaceutical Sciences, Naresuan University, Phitsanulok 65000, Thailand; nattakanwadeek@nu.ac.th; 2 Agricultural Biochemistry Research Unit, Faculty of Sciences and Agricultural Technology, Rajamangala University of Technology Lanna Lampang, Lampang 52000, Thailand; tanongsaks@rmutl.ac.th; 3 Department of Pharmaceutical Chemistry, Faculty of Pharmaceutical Sciences, Prince of Songkla University, Songkhla 90112, Thailand; krit@pharmacy.psu.ac.th; 4 Department of Medical Technology, Faculty of Associated Medical Sciences, Chiang Mai University, Chiang Mai 50200, Thailand; songyot.anuch@cmu.ac.th; 5 Research Center of Pharmaceutical Nanotechnology, Faculty of Pharmacy, Chiang Mai University, Chiang Mai 50200, Thailand; 6 Department of Pharmaceutical Sciences, Faculty of Pharmacy, Chiang Mai University, Chiang Mai 50200, Thailand

**Keywords:** chrysin, aromatase inhibitor, pluronic, polymeric micelles, flavonoid, male ratio, sex-reversal

## Abstract

Siamese fighting fish (*Betta splendens*) are freshwater fish that are commonly found in Thailand and other Southeast Asian countries. In the present study, chrysin-loaded polymeric micelles (CPs) were developed and investigated for the masculinizing effects, survival rate, growth indices, and toxicity on Siamese fighting fish. CPs were prepared using a poloxamer. The micelle system of CPs that were formed at a chrysin-to-polymer ratio of 1:2 was found to be the most suitable monodispersed system and exhibited a nanosized diameter (74.2 ± 1.6 nm) with a narrow size distribution (0.288 ± 0.012). In vivo studies were performed using Siamese fighting fish larvae as animal models. In the in vivo toxicity study, the fish larvae were immersed in aqueous systems containing CPs that had five different chrysin concentrations of 1, 10, 100, 1000, and 10,000 ng/mL for 24, 48, and 72 h. Blank polymeric micelles and water were used as controls. The in vivo masculinization effect of CPs with different chrysin concentrations on the fish larvae was evaluated after 5 weeks of exposure. The results demonstrated that CPs with a chrysin concentration of 1000 ng/mL showed a masculinization effect of 94.59 ± 2.76% with a high fish larvae survival rate of 72.45 ± 5.09% and low toxicity. It was concluded that the developed CPs had a significant effect on the sex reversal of Siamese fighting fish larvae with a high survival rate.

## 1. Introduction

Fish species of the *Betta* genus are known for their fighting behavior. These fish fight with their kinds and others to protect their territories [1]. *Betta splendens* is a famous fighting freshwater fish in Thailand [2]. This species is known and widely accepted internationally as the “Siamese fighting fish” or “Siamese betta”. These fish have unique and beautiful characteristics. By nature, male Siamese fighting fish have a more beautiful body shape (long tail and fins) and are more colorful than females [3]. The price of male Siamese fighting fish individuals can even be four to ten times higher than that of females. Therefore, male Siamese fighting fish are cultured for sale and export. This is a business that generates a lot of income for farmers in Thailand [4,5]. Currently, obtaining male fish naturally is a problem because the proportion of male formation (approximately 40%) is always less than female formation. Accordingly, monosex male formation would be an advantage to breeders.

There are common techniques and methods of masculinization according to the sex cell orientation of an organism from female to male. The most common technique is to give steroid hormones to undifferentiated fish. Steroid hormones play an important role in sex direction by stimulating sex hormones in male and female organisms during gonadal differentiation in nonmammalian vertebrates [6,7]. Several studies report the masculinization of Siamese fighting fish using the dietary administration of synthetic steroid hormones [8,9]. Nevertheless, synthetic steroid hormones can cause environmental and public health problems [10]. Therefore, searching for a better fish masculinization technique is an important issue to be addressed.

**Figure 1:**
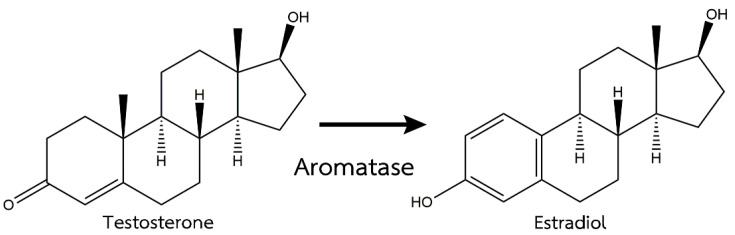
Mechanism of action of the aromatase enzyme [16].

In the last several years, fish masculinization with an aromatase inhibitor has been used in aquaculture. Aromatase is involved in sex differentiation and ovarian development via the estrogen biosynthesis pathway in many animal species [11,12]. Aromatase is an enzyme complex that plays an important role in converting androgens to estrogens with CYP19a and CYP19b expressions [13], as shown in Figure 1. Therefore, the inhibition of the aromatase enzyme can enhance androgen production. A previous study reported the effects of fadrozole, an aromatase inhibitor, on the androgen-induced masculinization of Japanese flounder (*Paralichthys olivaceus*) and showed the downregulation of CYP19a after the treatment [14]. Therefore, the lack of estrogen synthesis from the effects of aromatase inhibitors results in more androgens that are responsible for the differentiation of the testicles [15].

Chrysin, a natural flavonoid, is recognized as a potent aromatase inhibitor [17,18]. Honey, propolis, many plants, and even mushrooms are sources of chrysin [19,20]. In addition, chrysin was reported to be abundant in the fruit of *Oroxylum indicum* [21]. Previous studies have reported on several biological properties of chrysin, such as its antioxidant, anti-inflammatory, anticancer [20], and anti-neurodegenerative activities [22]. However, the low water solubility of chrysin causes limitations in its application.

Nanodelivery systems currently receive increasing interest and are popular approaches for solubilizing and stabilizing water-insoluble active ingredients, as well as enhancing their bioavailability [23]. A polymeric micelle is one of the attractive nanodelivery systems due to its simplicity of fabrication and high stability [24]. Polymeric micelles are self-assembling nano-constructs of amphiphilic polymers or copolymers. They show high benefits in medicinal and pharmaceutical applications for the delivery of various active agents [25,26]. They have received immense popularity due to their biocompatibility and capacity to solubilize poorly soluble pharmaceutical agents [27,28,29]. A poloxamer (a biocompatible polymer) is one of the suitable polymers for the potential dissolution of hydrophobic active compounds since it can improve the aqueous solubility, stability, and bioavailability of the drugs [30,31]. Generally, the hydrophobic compounds can be entrapped in the hydrophobic core of the poloxamer micelles and the whole system can be soluble in water. In previous studies, chrysin was used as a masculinization agent in zebrafish [32]. However, as far as we know, there have been no reports on the use of chrysin to masculinize Siamese fighting fish. This might be one of the first studies using this compound in the masculinization of *Betta* species.

The aim of the present study was to investigate the masculinization effects of polymeric micelles entrapping chrysin that were developed for male formation in Siamese fighting fish. In addition, the effects on the survival rate, growth indices, and toxicity to the fish were also evaluated.

## 2. Materials and Methods

### 2.1. Materials

Chrysin and polyoxyethylene 20 sorbitan monooleate (Tween 80) were purchased from Sigma-Aldrich, St. Louis, MO, USA. Absolute ethanol was purchased from Merck Millipore (Darmstadt, Germany). *Moina siamensis* cysts were purchased from the ornamental fish market in Lampang, Thailand. Pluronic F-127, a trade name of poloxamer 407, was from BASF Ludwigshafen, Germany. Nylon filter membrane was purchased from Anpel Laboratory Technologies (Shanghai) Inc. (Shanghai, Chaina). Milli-Q water was obtained from a Milli-Q water purification system manufactured by Millipore (Billerica, MA, USA). All solvents and other materials were of analytical grade.

### 2.2. Fish and Rearing Conditions

The mature Siamese fighting fish males (weight 2.00 ± 0.42 g and total length 7.54 ± 0.55 cm) and females (weight 1.08 ± 0.37 g and total length 4.74 ± 0.46 cm) were purchased from the ornamental fish market in Lampang, Thailand. They were individually acclimated to laboratory conditions in aquaria of 2 L with dechlorinated tap water (at 25 °C, pH 7.54, total hardness 118 ppm, alkalinity 101 ppm) and fed twice a day with *M. siamensis* cysts for 2 weeks. One female inside a floating transparent plastic shelter was placed in each male aquarium to familiarize them and stimulate the release of pheromones for spawning. After the bubble nest formation by a male spawner, the shelter was removed to allow them to start mating. After finishing the process of spawning, the female was moved out from the aquaria. The fertilized eggs were facilitated by oxygenation and attended by the male spawner. The hatching took place within 24–48 h; then, the male spawner was removed. After the yolk-sac was absorbed, the 4 days post hatch (dph) larvae were transferred to a new container with aeration, held in natural light conditions, and started with twice-daily feeding with fish meal powder for 4 weeks before the in vivo toxicity and masculinization experiments. The methods of these experiments were approved by the Naresuan University Animal Care and Use Committee (NUACUC), protocol number NU-AQ 600710. All experiments were performed in accordance with relevant guidelines and regulations.

### 2.3. Development of the Polymeric Micelles Containing Chrysin

Chrysin-loaded polymeric micelles (CPs) were prepared according to a method that was previously described [32]. Briefly, chrysin was dissolved in ethanol at a ratio of 1:1 (*w*/*v*). The obtained chrysin solution was added dropwise with Pluronic F-127 solution to obtain various mixtures containing chrysin-to-polymer weight ratios of 1:1, 1:2, 1:3, 1:4, 1:5, 1:10, and 1:15. After that, a proper amount of Tween 80 (approximately 10% of the chrysin) was added. Subsequently, deionized water was added to the mixture to obtain a water content of approximately 10 times that of the amount of chrysin. The obtained mixtures were centrifuged at 12,000× *g* for 10 min and filtered through a 0.22 μm nylon membrane (13 mm diameter) to remove the non-encapsulated precipitated chrysin. CPs were obtained in the filtrates. The filtrates were frozen at −20 °C and subsequently lyophilized under vacuum for 24 h. The lyophilized CPs were kept in the refrigerator for further use. To obtain the desired final concentration of chrysin for the in vivo studies, a sufficient amount of the lyophilized product was rehydrated in deionized water and vigorously mixed using a vortex mixer for 10 min at room temperature. The concentration of chrysin in the rehydrated stock CP solution was determined using high-performance liquid chromatography (HPLC). The blank polymeric micelles (blank PMs) were prepared according to the same protocol without chrysin.

### 2.4. HPLC Analysis

The chrysin concentration of the rehydrated stock solution was quantitated on an Agilent 1260 Infinity HPLC system (Agilent Technologies, Inc., Waldbronn, Germany). The separation was done using Hypersil™ ODS C18 HPLC Columns (4.0 mm × 250 mm, 5 μm) (Agilent Technologies, Inc., Santa Clara, CA, USA). A mixture composed of methanol and 1 M acetic acid in water (80:20) was used as a mobile phase. The separation was performed by injecting 10 μL of the sample with a flow rate of 1.0 mL/min at 25 °C and detected at 270 nm. The chrysin content was calculated from the equation of the chrysin standard curve in the concentration range of 31.25–500.00 μg/mL.

### 2.5. Characterization of the CPs

The lyophilized CPs were diluted with Milli-Q water at a weight ratio of 1:100 (CPs:water). The average droplet size and size distribution of the obtained CPs were investigated via dynamic light scattering using photon correlation spectroscopy (PCS) with Zetasizer version 7.03 software (Zetasizer Nano ZS, Malvern Instruments Ltd., Worcestershire, UK). Measurements were carried out at a fixed angle of 173° at 25 °C. The experiments were performed in triplicate with at least three independent experiments. Among the developed CPs, those with a small droplet size and narrow size distribution were selected for trials in the in vivo studies; see Table 1 and the corresponding results.

### 2.6. In Vivo Toxicity Study

An in vivo toxicity assessment was performed using 140 gender-undifferentiated Siamese fighting fish larvae per experiment. The fish larvae were divided into seven groups. Each group (*n* = 20) was placed into 6 mL of water in 60 mm diameter glass Petri dishes (Petriq, Darmstadt, Germany). Each sample group was added with the selected CPs rehydrated stock solution to obtain final chrysin concentrations of 1, 10, 100, 1000, and 10,000 ng/mL in glass Petri dishes containing 10 mL of dechlorinated tap water. Blank PMs and pure water were used as the vehicle and negative control, respectively. In addition, the systems were maintained at 26 ± 2 °C throughout the experiment. The mortality of the fish larvae after exposure to different concentrations was observed at 24, 48, and 72 h under a stereomicroscope (Nikon, Tokyo, Japan). The experiments were carried out with three independent replications. At the end of the experimental period, the fish that survived were maintained in a laboratory until they were in a healthy condition and then were donated to the aquarist as pet fish.

### 2.7. In Vivo Masculinization Effect

In this study, 120 gender-undifferentiated Siamese fighting fish larvae were used per experiment. The larvae were divided into 6 groups. Each group (*n* = 20) was placed into 6 mL of water in 60 mm diameter glass Petri dishes. Each sample group was subjected to the selected rehydrated CP stock solution to obtain final chrysin concentrations of 1, 10, 100, and 1000 ng/mL in glass aquaria (150 × 150 × 225 mm) containing 2 L of dechlorinated tap water. The other two groups were exposed to blank PMs and pure water as the vehicle and negative control, respectively. The water system was maintained at 26 ± 2 °C throughout the experiment. The treatment media in the glass aquaria were changed every 48 h by replacing the media with fresh preparations of treatment media. The water quality was checked before each treatment media replacement. The fish larvae were fed three times daily and held under natural light conditions (light/dark cycle of 14/10 h). After 5 weeks of treatment, the sex ratio, expressed as a male ratio percentage, and the survival rate (SR) of the fish were evaluated using Equations (1) and (2), respectively:

Male ratio (%) = (MF/TF) × 100,(1)

SR (%) = (TF/TFb) × 100,(2)

where MF and TF are the number of male fish and total fish (male and female) at the end of the experiment, and TFb is the total number of the fish (male and female) at the beginning of the experiment.

**Figure 2:**
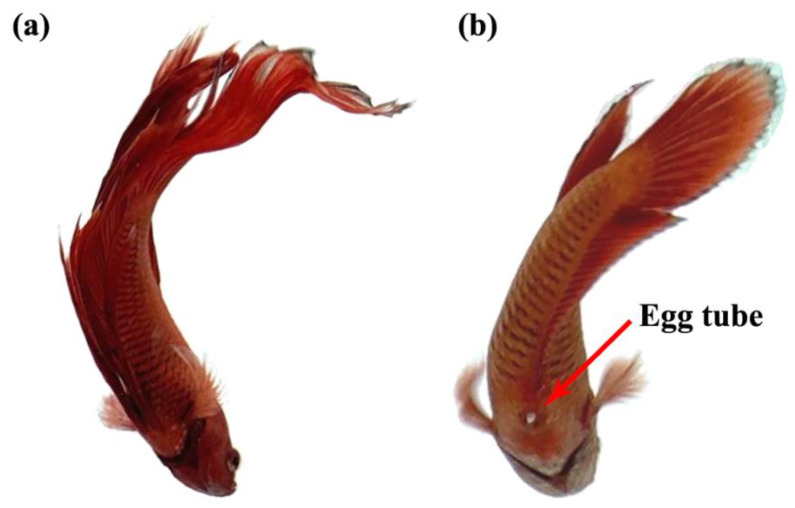
The anatomy of male (**a**) and female (**b**) of Siamese fighting fish.

An important criterion that is used to distinguish the sexes of Siamese fighting fish is the visibility of an ovipositor or egg tube in the female, as shown by the red arrow in Figure 2. In addition, the body size and shape, fin size, gill cover, color pattern, and behavior were also used to distinguish the sexes [33].

In addition, the weight and total length of each fish were measured weekly after hatching using a digital balance and digital vernier caliper, respectively. The experiments were carried out with three independent replications. At the end of the experiment period, the fish that survived were maintained in a laboratory until they were in a healthy condition and then they were donated to the aquarist as pet fish.

### 2.8. Statistical Analysis

The data are presented as the mean ± standard error of the mean (S.E.M.) from three independent experiments; the normality of the data was checked using Kolmogorov–Smirnov’s test, skewness, and kurtosis. Variance homogeneity was determined using Levene’s test. The ANOVA followed by Tukey’s post hoc test was used to analyze the data of CP characterization, in vivo toxicity, and in vivo masculinization studies. The probability values less than 0.05 (*p* < 0.05) were considered statistically significant.

## 3. Results and Discussion

### 3.1. Development and Characterization of CPs

Pluronic F-127 is a polyoxyethylene-polyoxypropylene surface-active block polymer. It is widely employed for drug delivery. It was used in this study because of its unique properties to form desirable polymeric micelles with high drug entrapment efficiency. In our preliminary experiment, chrysin was loaded into the micelles of this polymer. The results showed that the obtained CP system clearly indicated that high solubility of chrysin entrapped in the polymeric micelles was immediately obtained. However, precipitation of the drug was observed after a few minutes at room temperature (data not shown). Recently, an interesting approach was reported regarding the adoption of mixed micelles for the enhancement and overcoming of some disadvantages of the polymeric micelles [34]. The mixed micelles of Pluronic F-127 with certain surfactants, such as Cremophor EL and Tween 80, were reported to enhance the water solubility of norfloxacin and valsartan, respectively [35,36]. Therefore, in the present study, Tween 80 was used to form mixed micelles of Pluronic F-127 for entrapping chrysin. The obtained system was clear, and no drug precipitation was observed, even after being left for a week in a closed container at room temperature. The samples after lyophilization were in a semisolid form akin to a gel. After diluting with water, the blank PMs showed a clear and colorless solution, whereas the obtained CP system demonstrated a clear yellowish solution. These results indicated that chrysin was successfully loaded in the poloxamer mixed micelles. In addition, all developed CPs showed systems without any precipitation of chrysin, even after being kept at room temperature for more than 30 days. The average size and size distribution of the CPs and blank PMs are presented in Table 1.

It was found that loading chrysin in the polymeric micelles caused enlargement of the micelles. The average particle size of the prepared CPs was in a range of 68.3 ± 2.0–84.7 ± 1.2 nm, whereas that of the blank PMs was 21.3 ± 0.8 nm. The particle sizes of CPs of all ratios were obviously larger than that of the blank PMs. In addition, it was found that increasing the polymer content decreased the size of the micelles. The particle size distribution is one of the important parameters for the evaluation of nanodelivery systems. It is a parameter that is used to define the size range of the obtained particles and is commonly expressed as the “polydispersity index” (PdI). This parameter can describe the degree of non-uniformity of the obtained particles. A narrow size distribution indicates that the obtained particles are uniform, whereas a larger PdI value indicates a polydisperse system. The results of the present study showed that the PdI values of the CPs were slightly higher than that of the blank PMs. Most of the CPs showed a PdI in the range of 0.250–0.388. PdI values in the 0.2–0.3 range are acceptable for pharmaceutical applications. This is because such a preparation contains a homogenous population of nanocarriers [37]. It was considered that the system of CPs at a chrysin-to-Pluronic-F-127 ratio of 1:2 was the most suitable formulation for further study due to the appropriate mean size (less than 100 nm) and narrow distribution (PdI < 0.300). There was no significant difference in the mean size and PdI between the CPs in the 1:2 ratio and other ratios with more polymer content (1:3, 1:4, 1:5, 1:10, and 1:15). Therefore, this system was selected for further experimentation.

### 3.2. In Vivo Toxicity of the Developed CPs

Nontoxicity to fish is one of the important criteria for ideal fish masculinization. The masculinizing agent at the used concentration should be safe for fish. In general, the concentration of a test sample that causes mortality of 20% or less is regarded as safe [38,39]. In the current study, the toxicity of the selected CPs in larvae of Siamese fighting fish was investigated. The results are presented in Table 2.

It was found that the percentage of fish larvae mortality increased with an increased concentration of chrysin. However, the systems with a chrysin concentration of less than 100 ng/mL were regarded as safe to the fish larvae because the mortality of these systems throughout the experimental period was less than 20%. The results also demonstrated that the fish larvae could safely be in contact with the CPs that had a chrysin concentration of 1000 ng/mL for 48 h as the mortality was not more than 20%. Further exposure to 72 h led to a slight increase in mortality to 26%. According to a previous study [32], toxicity was classified as moderate to severe when the mortality was 30–100%. Therefore, we considered that mortality of less than 30% could be classified as mild toxicity (10–30% mortality). No mortality of the fish larvae was observed after transferring to the clean media and monitoring for one week. It was concluded that the toxicity of chrysin in the CP systems to the fish larvae was dose- and time-dependent. There was no significant difference in fish larvae mortality (*p* < 0.05) between the four different concentrations of chrysin (1, 10, 100, 1000 ng/mL) for 48 h of exposure, as well as for blank PMs and water. Therefore, these systems were selected for further study. The CP system with a chrysin concentration of 10,000 ng/mL was not selected because it was classified as a system that displayed moderate-to-severe toxicity to fish larvae (30–100% mortality).

### 3.3. In Vivo Masculinization Effect of the Developed CPs

**Figure 3:**
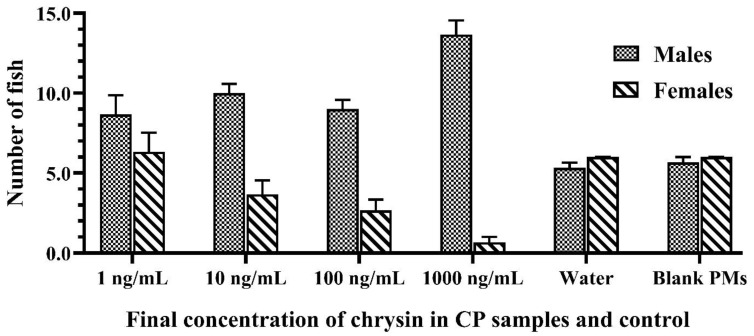
The number of Siamese fighting fish larvae that were exposed to 1–1000 ng/mL of chrysin in CPs, blank PMs, and water at the end of the experiment. Data are presented as the mean ± S.E.M.

The sex change of Siamese fighting fish depends on the presence of steroid hormones in the early development of the animal [40]. Chrysin acts as an aromatase inhibitor where it can inhibit the action of the aromatase enzyme involved in the process of estrogen production. Thus, the formation or level of the testosterone hormone was relatively increased and promoted the formation of male genital organs. The main factors that affect the success of sex reversal in fish are the applied protocol and the appropriate drug [41,42]. In the present study, the developed CPs showed a masculinization effect on Siamese fighting fish larvae in a dose-dependent manner, as the results show in Table 3 and Figure 3.

**Figure 4:**
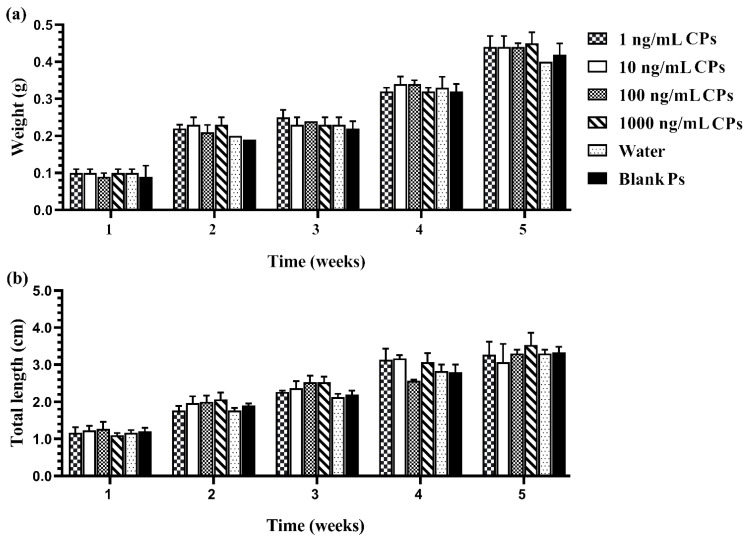
Growth performance: weight (**a**) and total length (**b**) of Siamese fighting fish larvae that were exposed to CPs with different chrysin concentrations, blank PMs, and water for 1–5 weeks. Data are presented as the mean ± S.E.M. (*p* < 0.05).

The results also demonstrated that sex reversal due to the developed CPs was significantly higher than the negative and vehicle controls (*p* < 0.05). There was no significant difference in the growth indices of fish larvae caused by different concentrations of chrysin in the CPs and the control groups, as shown in Figure 4. The growth performance, weight, and total length of the fish that survived in each treated group increased every week during monitoring. External factors, such as the water quality, the quantity of feed, and improper handling, also influence the survivability of the fish. Furthermore, water quality and environmental cues have an effect on fish masculinization. Inappropriate temperature, pH, ammonia or nitrate levels, and relative density can cause stress to the fish, resulting in measurement error [43,44]. Therefore, water quality and environmental cues in the present study were checked and evaluated according to the criteria of water quality control. The water quality in each aquarium during this experiment was monitored and controlled as follows: temperature 25–26 °C, pH 7.9–8.4, total hardness 120–160 ppm, alkalinity 90–96 ppm, total ammonia nitrogen level 0.1–0.2 ppm, and NH_3_ 0.01–0.015 ppm.

All developed CPs were found to be effective at inducing male ratios to more than 50%, even at low doses. The CPs containing the highest chrysin concentration (1000 ng/mL) produced 94.59 ± 2.76% males with an SR of 72.45 ± 5.09%. Hence, the masculinization of Siamese fighting fish larvae with developed CPs consisting of 1000 ng/mL chrysin was successful and is recommended to aquarists to increase the male ratio of *B. splendens*. In previous studies, successful male formation of Siamese fighting fish larvae (80–100% male ratio) using a honey solution composed of 5 mL of honey in 1 L of water was reported [45,46]. However, the SR of the fish exposed to the honey solution was less than 50% at the end of their studies. Furthermore, honey was reported to exhibit masculinization of juvenile red claw crayfish with an 80% male ratio and a 70% SR [47]. As chrysin is a compound that is commonly found in honey, this result revealed the potential of chrysin as a masculinizing agent to other fish species. Therefore, the developed CPs have the potential to be applied for male formation in other fish species. In addition, there was no significant difference in the SR between different concentrations of chrysin in CPs and the control groups. The SR of the fish that were exposed to the developed CPs was higher than that of the control groups. Since chrysin is a potent aromatase inhibitor [16,17,18], the level of testosterone in the fish that were exposed to CPs was likely higher than that of the control groups. It is known that testosterone regulates several physiological mechanisms that promote fish growth, such as protein synthesis and maintaining muscle mass and bone formation [48]. Moreover, chrysin showed an immunostimulant effect [49].

Furthermore, both chrysin and the poloxamer are safe for the environment and animals. Pluronic F-127 is one of the biodegradable polymers; thus, it is safe to be used [50,51]. In the case of chrysin, it is a natural flavonoid and was reported to possess several biological activities. Moreover, chrysin was reported to prevent tissue damage by affecting oxidation via anti-inflammatory and anti-oxidative effects of acute toxicity of rainbow trout [51]. However, it was reported that it demonstrated cell toxicity and inhibition of DNA synthesis at very low (2 μM) concentrations in a normal trout liver cell line [52]. Therefore, the masculinization of fish larvae with developed CPs needs further molecular biology toxicity studies.

## 4. Conclusions

In the present study, CPs were successfully developed using mixed micelles of Pluronic F-127 and Tween 80. The formulated CPs could effectively improve the water solubility of chrysin permanently without any sign of precipitation, even after a long storage duration at room temperature. The composition ratio of chrysin and Pluronic F-127 affected the size and size distribution of the obtained CPs. The system of CPs that was obtained from a chrysin-to-polymer ratio of 1:2 was suitable for further masculinization studies due to the appropriate mean size and narrow distribution. The CPs with a chrysin concentration of 1000 ng/mL induced 95% masculinization with a high survival rate of the fish larvae. The suitable CP systems containing chrysin concentrations up to 1000 ng/mL are recommended for aquarists to increase the male ratio of Siamese fighting fish. The masculinization technique presented in this study can be applied to other fish species.

## Figures and Tables

**Table 1:** Particle size and size distribution of the CP samples at various chrysin-to-polymer ratios.

CP Samples and Control	Particle Size (nm)	Size Distribution
Blank PMs	21.3 ± 0.8 a	0.218 ± 0.011 a
1:1	84.7 ± 1.2 b	0.420 ± 0.018 b
1:2	74.2 ± 1.6 c	0.288 ± 0.012 c
1:3	72.5 ± 2.2 c	0.322 ± 0.019 cd
1:4	72.4 ± 2.1 c	0.286 ± 0.013 c
1:5	70.0 ± 2.2 c	0.279 ± 0.014 ac
1:10	68.9 ± 2.6 c	0.256 ± 0.012 ac
1:15	68.3 ± 2.0 c	0.250 ± 0.019 ac

Data are presented as the mean ± S.E.M. of three independent replicates. Different letters indicate significant differences between treatment groups (*p* < 0.05).

**Table 2:** Mortality of the Siamese fighting fish larvae after exposure to CPs.

CP Samples and Control (Final Concentration of Chrysin (ng/mL))	Mortality (%) after Exposure Time		
	24 h	48 h	72 h
1	16.00 ± 4.62 a	16.00 ± 4.62 a	16.00 ± 4.62 a
10	9.33 ± 3.53 a	9.33 ± 3.53 a	9.33 ± 3.53 a
100	16.00 ± 2.31 a	16.00 ± 2.31 a	16.00 ± 2.31 a
1000	20.00 ± 2.31 a	20.00 ± 2.31 a	26.00 ± 2.31 b
10,000	65.33 ± 7.42 b	73.33 ± 7.42 b	90.67 ± 5.33 c
Water	9.33 ± 1.33 a	9.33 ± 1.33 a	9.33 ± 1.33 a
Blank PMs	9.33 ± 1.33 a	9.33 ± 1.33 a	9.33 ± 1.33 a

Data are presented as the mean ± S.E.M. of three independent replicates. Different letters indicate significant differences between treatment groups (*p* < 0.05).

**Table 3:** Effects of CPs on the male ratio and survival rate of Siamese fighting fish larvae after 5 weeks of maintenance.

CP Samples and Control (Final Concentration of Chrysin (ng/mL))	Male Ratio (%)	Survival Rate (%)
1	56.41 ± 6.41 a	74.51 ± 5.19 a
10	74.53 ± 4.03 b	68.53 ± 5.11 a
100	76.67 ± 3.33 b	56.86 ± 1.96 a
1000	94.59 ± 2.76 c	72.45 ± 5.09 a
Water	46.00 ± 1.73 a	56.31 ± 1.94 a
Blank PMs	47.62 ± 2.38 a	57.17 ± 1.66 a

Data are presented as the mean ± S.E.M. of three independent replicates. Different letters indicate significant differences between treatment groups (*p* < 0.05).
